# A Novel 2D Standard Cartesian Representation for the Human Sensorimotor Cortex

**DOI:** 10.1007/s12021-019-09441-y

**Published:** 2019-12-03

**Authors:** Mark L.C.M. Bruurmijn, Wouter Schellekens, Mathijs A.H. Raemaekers, Nick F. Ramsey

**Affiliations:** Brain Center Rudolf Magnus, University Medical Center Utrecht, Utrecht University, Heidelberglaan 100, PO Box 85500, 3508 GA Utrecht, The Netherlands

**Keywords:** Brain normalization, Sensorimotor cortex, Functional MRI, Somatotopic mapping

## Abstract

For some experimental approaches in brain imaging, the existing normalization techniques are not always sufficient. This may be the case if the anatomical shape of the region of interest varies substantially across subjects, or if one needs to compare the left and right hemisphere in the same subject. Here we propose a new standard representation, building upon existing normalization methods: Cgrid (Cartesian geometric representation with isometric dimensions). Cgrid is based on imposing a Cartesian grid over a cortical region of interest that is bounded by anatomical (atlas-based) landmarks. We applied this new representation to the sensorimotor cortex and we evaluated its performance by studying the similarity of activation patterns for hand, foot and tongue movements between subjects, and similarity between hemispheres within subjects. The Cgrid similarities were benchmarked against the similarities of activation patterns when transformed into standard MNI space using SPM, and to similarities from FreeSurfer’s surface-based normalization. For both between-subject and between-hemisphere comparisons, similarity scores in Cgrid were high, similar to those from FreeSurfer normalization and higher than similarity scores from SPM’s MNI normalization. This indicates that Cgrid allows for a straightforward way of representing and comparing sensorimotor activity patterns across subjects and between hemispheres of the same subjects.

## Introduction

In functional brain imaging (functional MRI; fMRI), spatial normalization is often applied, where scans are transformed into a common space, so that the same coordinates in different subjects correspond to the homologous anatomical location in the brain. This makes statistics at a group level possible, allowing for the comparison of brain activity patterns between groups of subjects, for example patients and healthy controls. The quality of the normalization is a central determinant of the quality of the group-level statistics (Pizzagalli et al. 2013), making accurate normalization a crucial part of the processing pipeline.

To join multiple brain images together for comparison of brain activation between groups (for example patients versus controls) or determining common areas of activation (mapping), several options are available and widely used. One is 3D normalization either using a single image (for example Talairach template), an average of co-registered images from multiple individuals unrelated to the study (for example MNI templates), or an average of study participants themselves (for example DARTEL (Ashburner 2007)). Alternatively, activity can be mapped on an inflated brain, where sulci are projected to a spherical surface or a flattened cortex map (Fischl et al. 1999), both of which allow for subsequent normalization (Qiu and Miller 2007; Van Essen et al. 2001).

For certain research questions, the existing techniques for representing brain activity patterns do not suffice, due to the fact that borders between regions (defined by gyral and sulcal patterns) reflect the natural 3D folding patterns of the brain (Pizzagalli et al. 2013). Some applications, for example a quantitative comparison of topographical mapping of sensory and motor functions, would benefit from a representation in the form of a 2D rectangular mesh. This constitutes an easy to interpret and uniform space, and would allow for easy comparison of activation patterns and distances between foci, while accounting for individual differences in the shape and size of sensorimotor cortex. Moreover, such a representation could make cross-hemispheric comparisons more direct and accurate, something which is not possible using existing normalization methods, as they typically do not conduct a registration of the two hemispheres. It also would accommodate a more direct comparison or combination of data from different studies.

A two-dimensional, grid-shaped representation has been described for the central sulcus, which was obtained by extraction of a 3D mesh of the central sulcus, which was subsequently reparametrized with the y axis along the direction of the central sulcus, and the x axis along the direction of the sulcal depth (Coulon et al. 2011). Although Coulon’s method elegantly maps the sulcus onto a grid, the sensorimotor cortex in fact extends also into the adjacent gyri, which is not included in their approach. Therefore, it is worthwhile transforming the whole pre- and postcentral gyrus into a Cartesian grid.

Here, we propose a novel extension to existing methods for standardization of regions in the human brain allowing for quantitative comparisons, which maps the whole gyri to a Cartesian grid: Cgrid (Cartesian geometric representation with isometric dimensions). Cgrid builds upon methods for inflating the cortex, and constitutes imposing a Cartesian grid on the region of interest using anatomical (atlas-based) landmarks. One brain region that seems particularly suitable for transforming into a rectangular mesh are the primary sensory and motor areas (S1 and M1), because of their more or less rectangular shapes with clear top, bottom and side boundaries. Cgrid is therefore first applied and validated on the precentral and postcentral gyrus. This special case is called ‘Cgrid-SMX’, where SMX stands for ‘sensorimotor cortex’.

Cgrid is meant to extend upon standard data preprocessing, and adding the possibility to easily compare patterns between subjects and between hemispheres. The presented implementation requires segmentation and atlas-based parcellation in FreeSurfer (Fischl 2012) and flat mapping with Caret (Van Essen et al. 2001), but accommodates any similar method.

The Cgrid-SMX mapping was evaluated using data from 20 healthy volunteers who each performed four motor tasks (moving left hand, right hand, feet, and tongue). As activation patterns for these basic motor tasks are expected to be similar across subjects, and within subjects across hemispheres, the similarities of the patterns of activity were calculated as a measure of validity of the transformation. The results were compared to the similarities obtained by SPM’s normalization to MNI space (a commonly used normal space) as well as to the similarity of activation patterns after FreeSurfer normalization. This was to provide a benchmark for the performance of our new method.

## Methods

### Subjects

Twenty healthy volunteers participated in this study (age 26.7 ± 8.8 years, 9 females, all right handed). Subjects had no history of neurological or psychiatric disorders. Data acquisition was approved by the medical-ethical committee of the University Medical Center Utrecht and all subjects gave their written informed consent in agreement with the declaration of Helsinki (World Medical Association 2013).

### MRI Data Acquisition and Analysis

MRI data were recorded using a Philips 3 T Ingenia system. A structural T1-weighted MRI image was acquired (TR/TE = 8.4/3.8 ms, voxel size: 1.00 × 1.00 × 1.00 mm^3^), followed by functional EPI images (TR/TE = 2500/39 ms, flip angle = 75°, axial orientation, FOV (AP, FH, LR) = 235 × 120 × 200 mm^3^, interleaved slice ordering, acquisition matrix 80 × 40 × 80, voxel size: 2.94 × 3.00 × 2.94 mm^3^). For data preprocessing, we used the software packages FreeSurfer (Fischl 2012), Caret (Van Essen et al. 2001) and SPM (Friston et al. 2007). Custom scripts for the Cgrid-SMX normalization were written in Matlab (The MathWorks Inc., Natick, MA) and IDL (Exelis Visual Information Solutions, Boulder, Colorado).

### Structural MRI Preprocessing

**Fig. 1:**
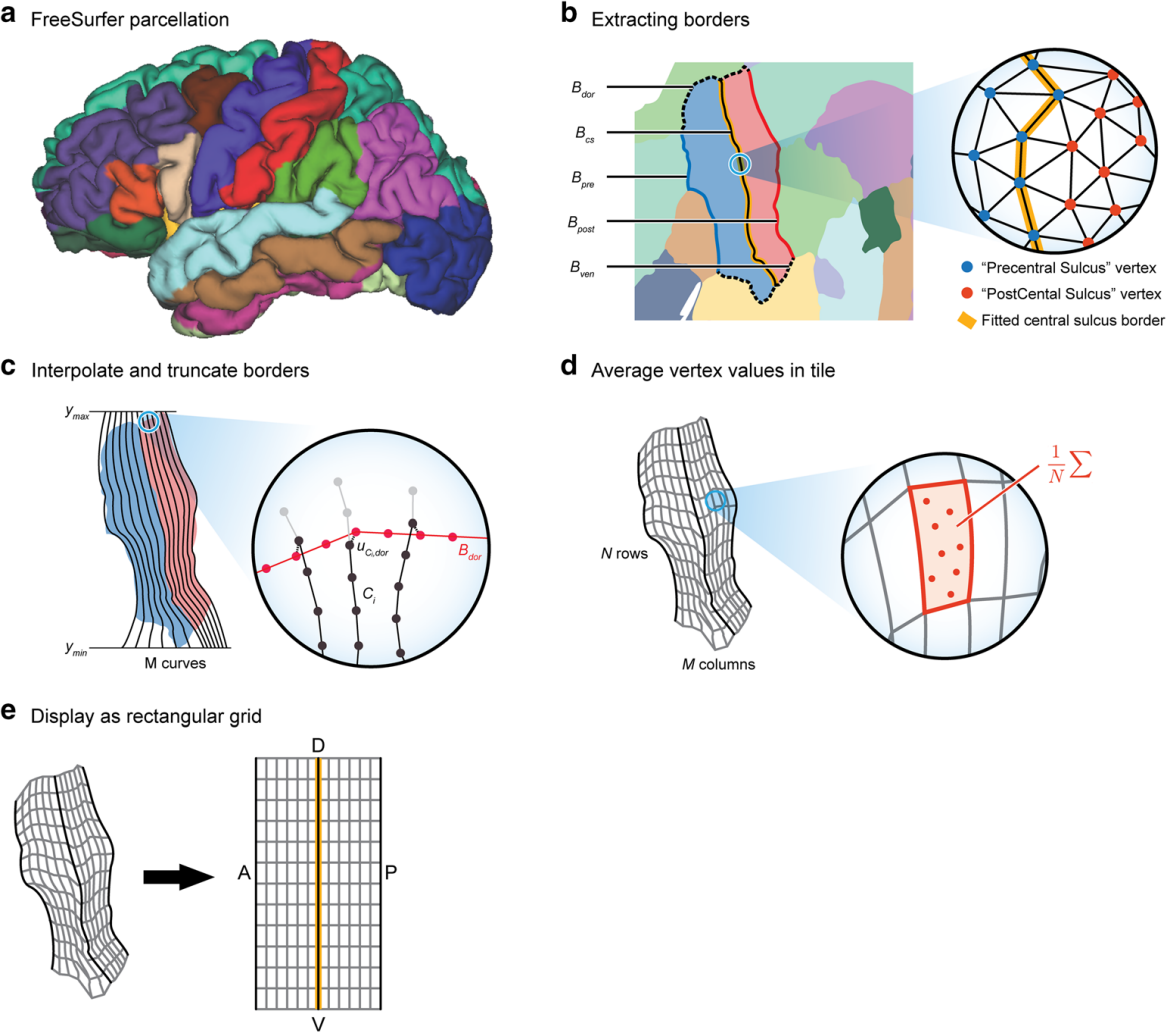
Applying Cgrid to the sensorimotor cortex. A: Brain parcellation from FreeSurfer. B: Flatmap representation, with the five borders that were extracted using labels from FreeSurfer’s cortical parcellation according to Eq. 2–6 (solid lines: “vertical borders” and dashed lines: “horizontal borders”). A vertex was considered to be part of a border if it had a neighboring vertex with another FreeSurfer label. C: 10th-order polynomials were fitted through the three vertical borders, and in-between vertical curves were created by interpolation between y_min and y_max. Each curve C_i was then truncated using the horizontal dorsal and ventral borders (drawn in red in the inset) by selecting the node points closest to any node on these horizontal borders. D: Truncated vertical curves were divided into vertical segments, resulting in N × M “tiles”. To map beta values from statistical maps to Cgrid, a beta value for each tile is calculated by averaging the beta values of vertices inside that tile. E: A Cgrid can be visualized as a rectangular grid, where the central sulcus is the middle, the anterior aspect (A) on the left side, posterior (P) on the right side, ventral (V) at the bottom and dorsal (D) at the top

For each subject, the cortical surface was reconstructed from the T1-weighted image using FreeSurfer, and automatically parcellated into ROIs using the Desikan-Killiany atlas (Desikan et al. 2006) (Fig. 1a). Each individual’s surface was then flattened using Caret, making sure that the central sulcus was oriented vertically (that is, dorsal aspect at the top, ventral aspect at the bottom, which is necessary for the Cgrid procedure).

### Definition of the Cgrid Standard Space

The flattened cortex was represented as a face-vertex mesh in 2D. Each vertex *v* has an x- and y-coordinate, *v*_*x*_ and *v*_*y*_. Notably, because a flat map is a deformation of a spherical surface, distances on the flat map will not exactly correspond to distances on the brain. Therefore we will consider distances on the flat map to be measured in arbitrary units (a.u.), although 1 a.u. will approximate 1 mm. Each vertex was tagged with the ROI label indicating the underlying Desikan-Killiany atlas region, and *L*(*v*) denotes the ROI label of vertex *v*. The topology describes which vertices are connected to form the faces of the mesh. Let the set of neighboring vertices of vertex *v* be denoted by Ω_*v*_.

The first step in defining the Cgrid standard space was the extraction of five anatomical borders. A border *B* between two ROIs was defined as the set of vertices having ROI label *L*_1_, while having one or more neighboring vertices with another ROI label *L*_2_:

1 $$ B\left({L}_1,{L}_2\right)=\left\{v|L(v)={L}_1,\exists w\in {\Omega}_v:L(w)={L}_2\right\} $$

Three ‘vertical borders’ (the central sulcus border *B*_*cs*_, the precentral sulcus border *B*_*pre*_ and the postcentral sulcus border *B*_*post*_, Fig. 1b) were defined using Eq. 1, where curly brackets indicate that *L*_2_ can be one of the given labels:

2 $$ {B}_{cs}=B\left(``\mathrm{Precentral}\ \mathrm{gyrus}",``\mathrm{Postcentral}\ \mathrm{gyrus}"\right) $$

3 $$ {B}_{pre}=B\left(``\mathrm{Precentral}\ \mathrm{gyrus}",\left\{``\mathrm{Pars}\ \mathrm{opercularis}",``\mathrm{Caudal}\ \mathrm{middle}\ \mathrm{frontal}",``\mathrm{Superior}\ \mathrm{frontal}"\right\}\right) $$

4 $$ {B}_{post}=B\left(``\mathrm{Postcentral}\ \mathrm{gyrus}",\left\{``\mathrm{SupraMarginal}",``\mathrm{SuperiorParietal}"\right\}\right) $$

Two ‘horizontal borders’ were defined, constraining the sensorimotor cortex at the dorsal (*B*_*dor*_) and ventral (*B*_*ven*_) side:

5 $$ {B}_{dor}=B\left(\left\{``\mathrm{PrecentralGyrus}",``\mathrm{PostcentralGyrus}"\right\},``\mathrm{ParacentralLobule}"\right) $$

6 $$ {B}_{ven}=B\left(\left\{``\mathrm{PrecentralGyrus}",``\mathrm{PostcentralGyrus}"\right\},``\mathrm{Insula}"\right) $$

The next step consisted of fitting a 10th order polynomial through each of the three vertical borders. The order 10 was chosen empirically and was found to result in a good balance between capturing the shape of the borders and still allowing for extrapolation, which is needed in a next step. For generating these fits, the vertical coordinate of the vertices (*v*_*y*_, the coordinate on the dorsal-ventral axis) was treated as the independent variable, and the horizontal coordinate (*v*_*x*_, the coordinate on the anterior-posterior axis) as the dependent variable. The vertical curves were resampled and extrapolated such that they ran from *y*_*min*_ to *y*_*max*_ in unit steps (arbitrary units), thereby making sure that they covered the whole sensorimotor cortex, where *y*_*min*_ and *y*_*max*_ were defined by:

7 $$ {y}_{min}=\min \left\{{v}_y|v\in {B}_{ven}\right\} $$

8 $$ {y}_{ma\mathrm{x}}=\max \left\{{v}_y|v\in {B}_{dor}\right\} $$

In-between vertical polynomial curves were then created by linear interpolation of each of the 11 polynomial coefficients regularly at *M* + 1 points, thereby effectively dividing the sensorimotor cortex into *M* “columns” (Fig. 1c).

As each in-between curve *C*_*i*_ ran from *y*_*min*_ and *y*_*max*_, some of them extended too far outside the sensorimotor cortex. Therefore, they needed to be truncated at the dorsal and ventral borders. Let the *X*_*i*_ nodes on the *i*th interpolated vertical curve *C*_*i*_ = {*u*_*j*_| *j* = 1. . *X*_*i*_}, *u* = (*u*_*x*_, *u*_*y*_). Ventral and dorsal cuts for curve *C*_*i*_ were defined as the nodes ${u}_{C_i, med}$ and ${u}_{C_i, dor}$ on the interpolated curves closest to any point on *B*_*ven*_ and *B*_*dor*_, where *d*(*u*, *v*) denotes the Euclidean distance between vertices (*v*) and nodes on the curve (*u*):

9 $$ {u}_{C_i, ven}=\underset{u\in {C}_i}{\mathrm{argmin}}d\left(u,v\right)\kern1.75em \forall v\in {B}_{ven} $$

10 $$ {u}_{C_i, dor}=\underset{u\in {C}_i}{\mathrm{argmin}}d\left(u,v\right)\kern1.75em \forall v\in {B}_{dor} $$

Each curve *C*_*i*_ was then divided into *N*_*rows*_ segments by resampling *C*_*i*_ from ${u}_{C_i, med}$ to ${u}_{C_i, dor}$ in 0.1 arbitrary unit steps. For this step, the length of each curve was first estimated by:

11 $$ {l}_i=\sum \limits_{j=1}^{X_i-1}d\left({u}_j,{u}_{j+1}\right) $$

Each of the vertical curves was then resampled again, where the distances between the nodes equaled *l*_*i*_/*N*. This resulted in a grid imposed on the sensorimotor cortex, consisting of *N* rows and *M* columns, denoted as *N* × *M* “tiles”.

The final step consisted of mapping all vertices from the cortical surface into the newly defined standard space, by treating each tile as a polygon and determining which vertices are enclosed by that polygon. As a result, each vertex was associated with one tile in Cgrid. This association allows for mapping any kind of MRI data to Cgrid space, for example anatomical data, such as cortical thickness, or functional data. This mapping consists of two steps: first, the MRI data needs to be projected onto the cortical surface reconstruction vertices (using tools from the FreeSurfer package). Second, per tile a value (thickness, functional beta, etc.) can be calculated by taking the mean of all vertices for that tile (Fig. 1d). In the Evaluation section, the mapping to Cgrid-SMX space is demonstrated with task-based functional data.

By convention, Cgrid visualizations in this paper are displayed (and processed) such that the precentral sulcus border is always on the left, and the postcentral border is always on the right. This means that the left half of the Cgrid images represents the precentral gyrus (M1), and the right part represents the postcentral gyrus (S1), regardless of the hemisphere (Fig. 1e).

## Evaluation

Task-based fMRI activation maps for the 20 subjects were mapped to Cgrid-SMX. Activation patterns were generated for four movement tasks (see ‘Task design’, below). Cgrid-SMX space was evaluated by calculating the within-subject (left-right) and between-subject similarities of activation patterns in Cgrid space. For this, a Pearson correlation between Cgrid-SMX activation patterns was used. To benchmark the results, Cgrid-SMX pattern similarities were then compared to within- and between-subject pattern similarities in MNI space from SPM. We focused on four regions of interest (ROIs): left M1, left S1, right M1, and right S1.

### Task Design

Subjects executed four separate movement tasks: following a visual cue, subjects were instructed to move their right hand (“Hand-Right task”, opening and closing), their left hand (“Hand-Left task”, opening and closing), their tongue (“Tongue task”, moving from left to right), or both feet (“Feet task”, rotating both feet about the ankle simultaneously). Each task was set up as a block design, with pseudorandom block durations ranging from 15 to 45 s followed by rest blocks ranging from 15 to 45 s.

### Cgrid Activation Maps

Task data was slice-time corrected, realigned and coregistered to the subject’s anatomical scan to correct for movements using SPM12 (http://www.fil.ion.ucl.ac.uk/spm/). A GLM analysis with one regressor for movement was applied to the task data using the contrast ‘movement versus baseline’, resulting in one statistical map (beta map) per task. These beta maps were then projected onto the cortical surface reconstruction vertices using FreeSurfer (with projection fraction 0.5 and a smoothing of 6 mm FWHM). A beta value was then computed per tile by taking the mean of the beta values for all vertices within that tile. This resulted in beta maps in Cgrid-SMX space for each of the four ROIs.

### MNI Activation Maps

To benchmark the performance of Cgrid space, functional scans were also normalized to MNI for all subjects using SPM12, and likewise smoothed with 6 mm FWHM Gaussian kernel. After normalization and smoothing, a GLM with one regressor for movement was fit to the task data and statistical maps were created using the contrast ‘movement versus baseline’.

Four ROI masks in MNI space (left M1, left S1, right M1, and right S1) were initially taken from the Brainnetome Atlas (Fan et al. 2016). Since the method of calculating similarities between hemispheres requires left and right ROIs to be symmetrical, the right M1 was flipped to the left hemisphere, and combined with left M1 (voxel-wise union). The resulting ROI was then flipped back to the right hemisphere. The same was done for S1. The resulting ROIs were used to mask the beta map and obtain activity patterns for the four tasks in each of the four ROIs.

### Within-Subject Pattern Similarity (Left-Right)

As the Cgrid-SMX space is expected to minimize anatomical differences between the left and right motor cortex, left and right activation patterns should demonstrate high similarity within subjects. For the Feet task and Tongue task, the similarity between left and right Cgrid patterns was calculated using Pearson correlation. For the hand tasks, the correlation between contralateral activation patterns was calculated, that is: the similarity between the left pattern from the Hand-Right task and the right pattern from the Hand-Left task. All Pearson correlations were transformed to ‘similarity (z-)scores’ using the Fisher z-transform (which is equal to the hyperbolic function arctanh), to allow averaging and statistical testing across subjects. The 6 similarity scores for each subject (Tongue, Hand and Feet for M1 and S1) were then averaged per subject over ROIs and tasks to obtain a single within-subject (left-right) similarity per subject for Cgrid. Similarity scores can be transformed back to (group-averaged) correlations using the inverse Fisher z-transform (the hyperbolic function tanh).

Similarity scores for MNI space were calculated similarly, and differences in similarity scores between Cgrid-SMX and MNI space were assessed using a paired-samples t-test.

### Between-Subject Pattern Similarity

To assess between-subject pattern similarity, a per-subject similarity score was calculated using a leave-one-out approach, where a pattern of the subject under investigation was correlated with the mean patterns of the other subjects. This resulted in similarity scores per task and ROI for every subject, which were then averaged to obtain a mean similarity score per subject. The same approach was applied to the patterns in MNI space, and a paired-samples t-test was conducted to compare the between-subject similarity scores for Cgrid and MNI space.

Since MNI is a 3D space and Cgrid is a 2D space, the differences in the dimensionality of the approaches might bias the performance. FreeSurfer includes surface based normalization through spherical registration, using the FS-average as template. All subjects were normalized using this approach. Then, activation patterns in FS-average space were extracted by selecting the beta values in the nodes of the pre- and postcentral gyrus. A between-subject similarity was calculated per subject following the same scheme as for the Cgrid and MNI, using a leave-one-out approach.

### Effect of Smoothing on between-Subject Correlations

For the within- and between-subject similarities, a Gaussian smoothing kernel of 6 mm FWHM was used. However, since the impact of a smoothing kernel can be different between Cgrid (2D space) and MNI space (3D), we tested the effect of the smoothing kernel on the similarities. This was done by repeating the between-subject analysis described above, using different smoothing kernels both in MNI space and on the cortical surface in the Cgrid pipeline (see above). Kernel sizes of 4, 6, 8, 10, 12, 18, 25 and 35 mm FWHM were used. A two-way repeated measures ANOVA was conducted to compare the effects of method and smoothing kernel size on the between-subject similarity score.

## Results

### Defining Cgrid Space

Surfaces reconstructions of all 20 subjects were generated using FreeSurfer. The five borders (central sulcus, precentral sulcus, postcentral sulcus, ventral border, and dorsal border) were extracted and visual inspection of the fitted curves confirmed that a 10th order polynomial fit was sufficient to capture the shape of the borders accurately in all subjects.

A Cgrid standard space was defined and resulted in a 28 × 84 tiled mesh per hemisphere in all subjects. A tile covered 2.62 ± 0.71 mm^2^ (mean ± sd) and contained 6 ± 1 vertices. On average, 21 ± 10 tiles (1.8% of all tiles) did not contain any vertices that were labelled as being part of the sensorimotor cortex; these tiles were mostly located at the edges of the Cgrid and were excluded from the correlation analyses.

### Mapping Beta Maps to Cgrid Space

**Fig. 2:**
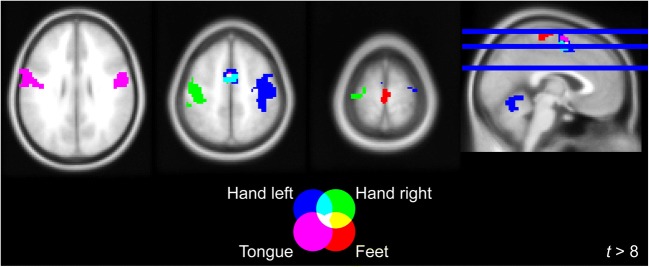
Group activation map of the movement tasks (contrasts used: Feet > baseline, Tongue > baseline, Left hand > baseline, and Right hand > baseline). Contrasts are displayed on a standard MNI brain with threshold t > 8

Volumetric statistical group maps of the tasks showed sensorimotor activation in distinctive foot, hand, and tongue areas (see Fig. 2). The feet and tongue tasks activated both the left and right sensorimotor cortex. There was no excessive motion (mean absolute translation over all subjects and tasks: 0.17 ± 0.10 mm; mean rotation: 2.8 × 10^−3^ ± 2.3 × 10^−3^ degrees).

**Fig. 3:**
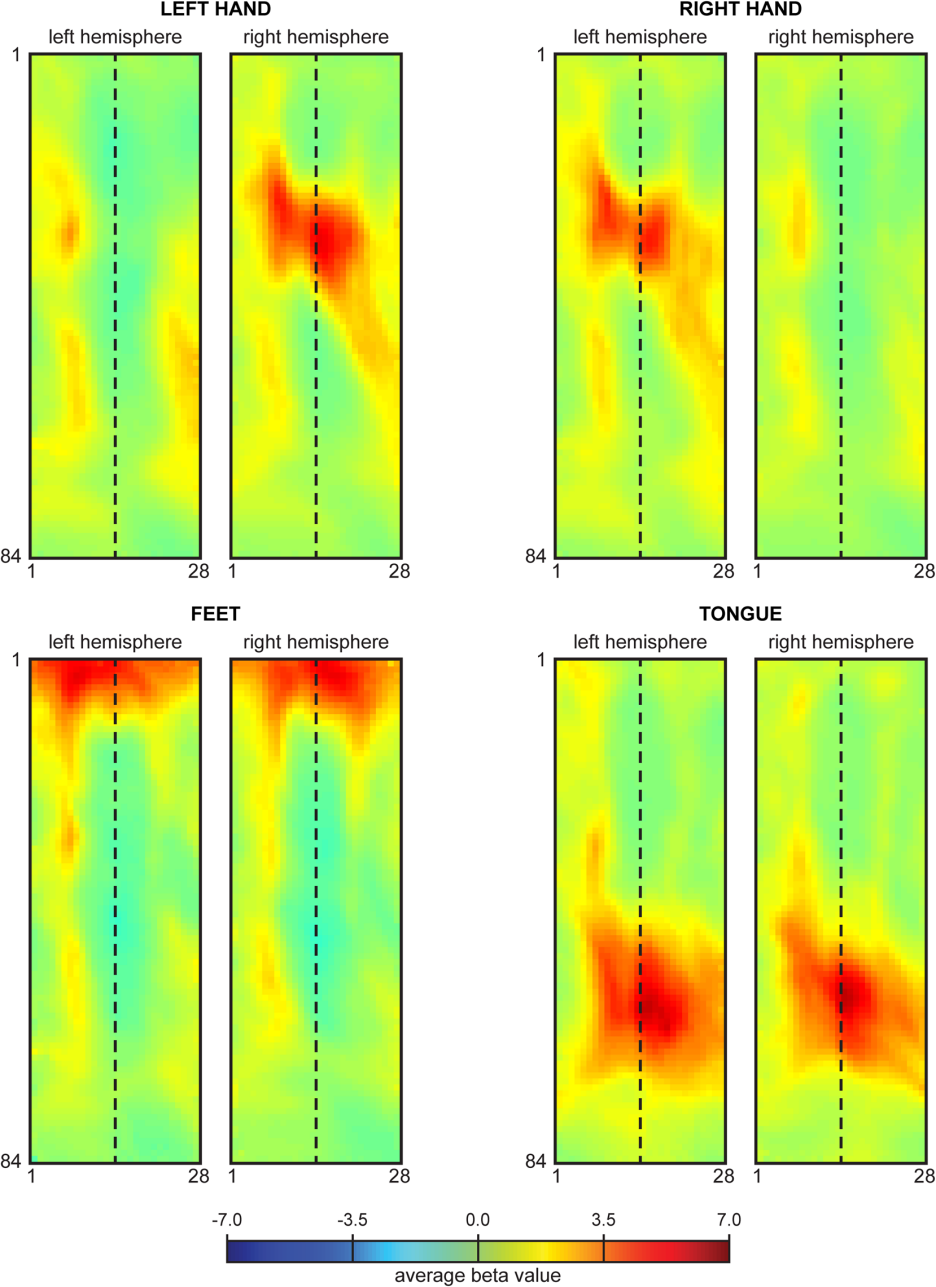
Beta maps in Cgrid, averaged over 20 subjects, for every task in both hemispheres. The dashed line indicates the central sulcus. The left border lies in the precentral sulcus, the right border on the postcentral sulcus (see fig. 1b). Note that for all Cgrid-SMXs the left side is anterior in the brain

**Fig. 4:**
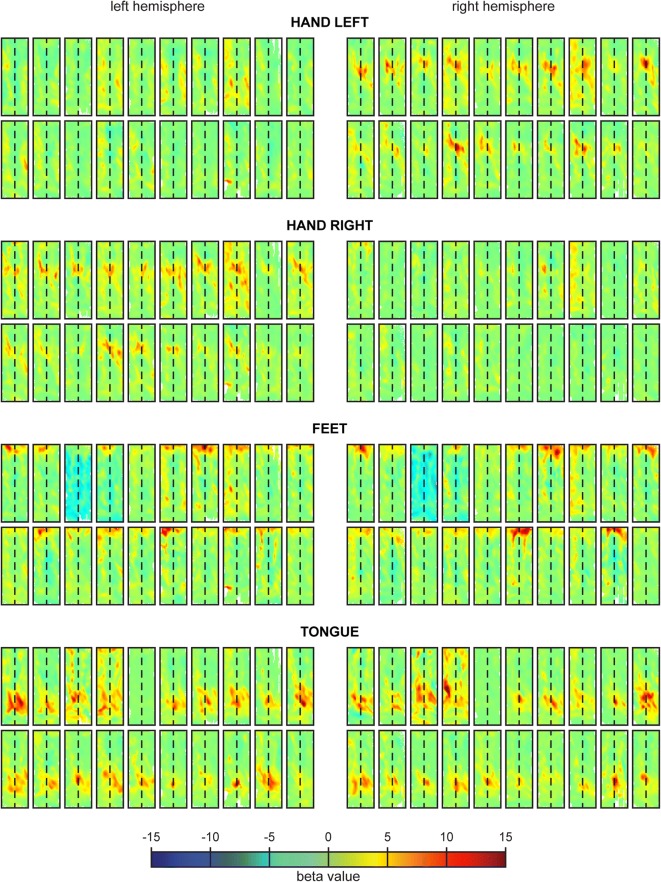
Beta maps in Cgrid for every subject (*N* = 20) and every task in both hemispheres

Visual inspection of the resulting Cgrid group-mean activation maps, averaged over subjects, confirmed that Cgrid was capable of capturing the different activation hotspot patterns associated with movement of the respective body parts (Fig. 3). Feet activation was located at the dorsal side of the sensorimotor cortex, tongue activation was located towards the ventral side, and hand activation was located mostly contralaterally at approximately 1/3 of the dorsal-ventral axis. Average activation hotspots for all tasks were mostly located within the central sulcus. Whereas the group average of Cgrid patterns demonstrated strong hotspot-like activation, task activation patterns per individual did not necessarily consist of only a single hotspot, but were sometimes complex patterns, varying somewhat across subjects (Fig. 4).

### Within-Subject Pattern Similarity (Left-Right)

**Fig. 5:**
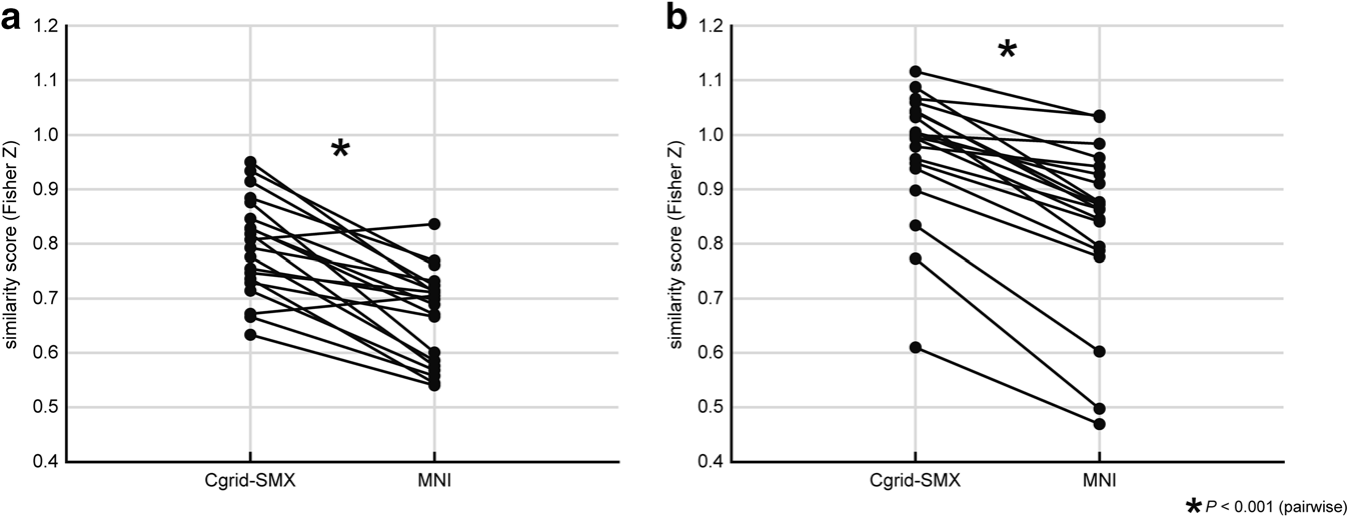
A: Within-subject similarities (averaged over tasks and hemispheres) per subject, for Cgrid (red dots) and MNI space (blue dots). Similarities in Cgrid space were significantly higher than in MNI space. B: Between-subject similarities (averaged over tasks and hemispheres) per subject, for Cgrid (red dots) and MNI space (blue dots). Similarities in Cgrid space were significantly higher than in MNI space

The similarities between left and right hemispheric patterns within subjects from feet, hand, and tongue tasks were computed using Fisher z-transformed Pearson correlations for both Cgrid and MNI space. A second-level paired t-test demonstrated a significantly higher similarity in Cgrid (Fisher Z = 0.80 ± 0.09, mean ± standard deviation) than in MNI space (Fisher Z = 0.67 ± 0.08); t(19) = 6.70, *p* < 0.001 (Fig. 5a).

### Between-Subject Pattern Similarity

The similarity of patterns between subjects was calculated per task and per ROI using Pearson correlations using a leave-one-out approach. A paired t-test demonstrated a significantly higher correlation in Cgrid (Fisher Z = 0.92 ± 0.09) than in MNI space (Fisher Z = 0.84 ± 0.16); t(19) = 8.25, p < 0.001 (Fig. 5b).

**Fig. 6:**
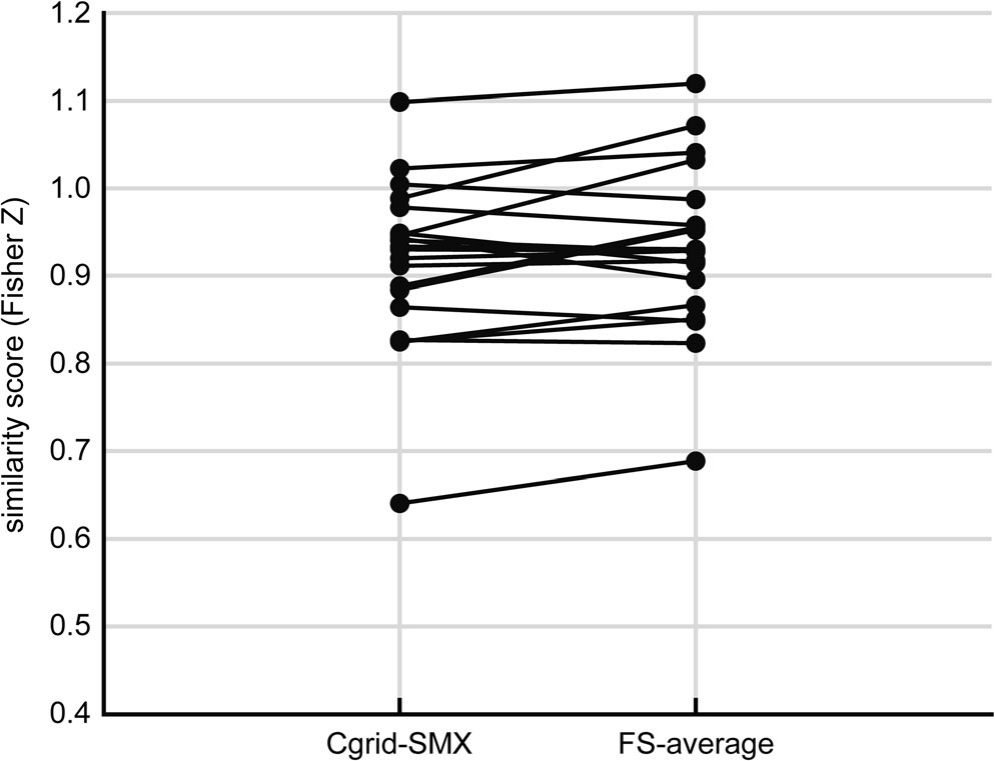
Between-subject similarities in Cgrid-SMX and in the FreeSurfer normalized space (FS-average). There was no significant difference in similarity scores between the two methods

Similarity scores were also calculated directly using FreeSurfer surfaces in averaged space (FS-average). There was no significant difference between similarity scores Cgrid and FS-average (Fisher Z = 0.93 ± 0.10); t(19) = −1.84, *p* = 0.082 (Fig. 6).

### Effect of Smoothing on between-Subject Correlations

**Fig. 7:**
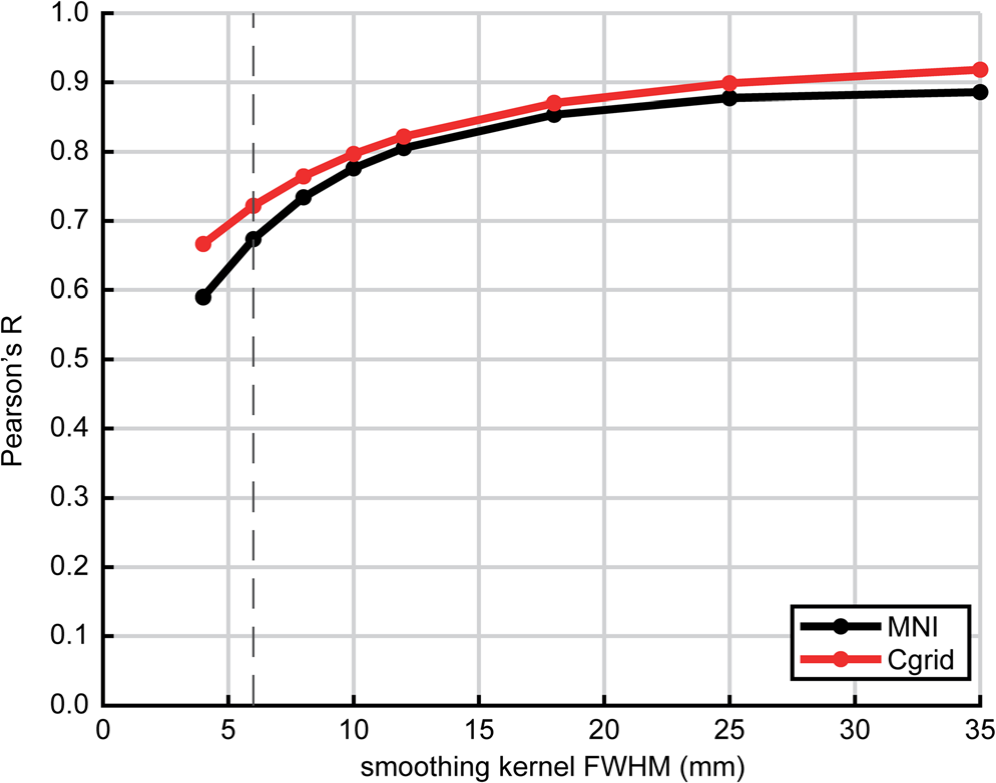
Between-subject correlations (averaged over tasks and ROIs) as a function of smoothing kernel size. The dashed line indicated the kernel size used for smoothing in both the Cgrid-SMX and MNI space analyses throughout the text (6 mm FWHM)

Calculating between-subject similarities with different smoothing kernels resulted in higher similarity scores with larger smoothing kernels for both Cgrid-SMX and MNI space (Fig. 7). A two-way repeated measures ANOVA showed a significant effect of method on the between-subject similarity score, indicating that Cgrid similarities are higher than similarities in MNI space for all smoothing kernel sizes.

## Discussion

We introduce Cgrid-SMX as a Cartesian representation of the sensorimotor cortex, based on anatomical atlas-based landmarks and building upon existing data processing methods. Cgrid imposes a grid on the sensorimotor areas, thereby effectively transforming them into a rectangular, tiled mesh. Cgrid was successfully applied to 20 healthy subjects on both the left and right hemisphere. Results of comparing sensorimotor activity patterns between individuals and between hemispheres yielded high similarity scores, exceeding those obtained with analysis of the same data in MNI space, but equal to similarity scores calculated in FreeSurfer space. Nevertheless, these findings indicate that Cgrid yields a representation that allows for a straightforward way of comparing activity patterns in sensorimotor cortex, which performs at least as good as representations from the more standard FreeSurfer and MNI approaches in terms of pattern similarities.

Transforming regions of the brain into a grid-like representation has also been reported in literature. It has been applied to the visual cortex, based on statistical modelling of the borders using visual stimuli (Corouge et al. 2004). Also the central sulcus has been transformed into a 2D grid mesh (Coulon et al. 2011), and even the whole cortex has been parametrized using the alignment of sulci (Auzias et al. 2013). However, there are some key differences between these approaches and Cgrid. First, the method described by Coulon only covers the cortex inside the central sulcus, whereas our method maps the surface of the whole gyrus. Second, the Cgrid method is described in such a way that it can be applied on any brain region, as long as clear borders can be defined. It does not statistically model the borders, but rather extracts them from existing atlases. This makes Cgrid a versatile tool, since it is easy to select a different set of borders if desired. Third, the simple geometry of the Cgrids allows for an easy to interpret visualization, which was one of the goals for the development of Cgrid.

The validity of using Cgrid was confirmed by multiple findings. First, analysis of Cgrid-transformed group-averaged activity patterns associated with movement (feet, left hand, right hand, and tongue) resulted in focal activation hotspots. The location of these hotspots allowed for a clear differentiation between the studied motor functions and preserved the topographical distinction between body parts, according to what is known from literature: feet activity was located near the medial wall, tongue activity was bilaterally located in the ventral sensorimotor area, and hand activation was located about halfway the dorsal-ventral axis, mainly on the contralateral hemisphere. Second, as Cgrid is designed as a representation accounting for anatomical differences, we expected a high similarity between the left and right Cgrid activation patterns within a subject, and also a high similarity between Cgrid activation patterns across subjects. Indeed, averaged over tasks and ROIs, similarity scores were high both for within- (Fisher Z = 0.8, corresponding to a Pearson correlation of R = 0.66) and between-subject (Fisher Z = 0.97, R = 0.75) comparisons. This indicates that there is a good correspondence of the functional localization in Cgrid both between left and right cortex within subjects, and between subjects, supporting the utility of the common space transformation. Finally, we compared the within-subject similarities and between-subject similarities of Cgrid activation patterns to those in MNI space, as this is the most widely used standard space for normalization.

When benchmarking Cgrid activity patterns against those from MNI, both within- and between-subject similarities were higher for Cgrid than for MNI space. It should be noted, however, that the comparison of these two methods should be taken with some caution. First, different spaces (2D flat map and 3D MNI volume) required the use of different atlases. The Desikan-Killiany atlas is provided with FreeSurfer and is the atlas from which borders for Cgrid are detected, but this atlas has been developed for surface-based analysis and can therefore not be used in 3D volumes. While a volumetric version of the Desikan-Killiany atlas exists, it only labels the grey matter voxels of the FreeSurfer average, rendering it unsuitable as an atlas for SPM volumetric normalization. Although the use of different atlases is not optimal, the labels used by these two different atlases (precentral and postcentral) indicate highly similar brain areas. Any difference in results that originates from differences in labels would be small. Second, although smoothing kernels with the same sizes were used in both Cgrid and MNI, the effect of smoothing may differ, as in Cgrid smoothing was done in 2D on the surface, and in MNI in 3D on the whole volume. Smoothing in 3D can possibly also include signals from for example white matter, or even from areas that are relatively remote when measures across the surface of the cortex, but proximate in 3D space. Comparison of the two normalization methods over a wide range of smoothing kernels, however, revealed that correlations were generally higher in Cgrid than in MNI space, even with larger kernels. Third, calculating a similarity between patterns from both hemispheres in MNI space was only possible when mirroring the masks for the somatosensory cortex across the longitudinal fissure. This is because for the correlation, left and right ROIs need to be symmetrical (with the same number of voxels and same spatial configuration), which is not necessarily the case in an atlas. Therefore, we mirrored the ROIs, although this does not yield an ROI that is perfectly anatomically aligned and possibly affects the correlation between left and right. Note that the limitation in this approach reflects one of the advantages of Cgrid, where coordinates within the left and the right hemisphere are automatically matched. Forth and finally, the comparison between MNI and Cgrid was performed only using the default settings for normalization in SPM12, and therefore indicate that Cgrid yields higher pattern similarities than normalization to MNI space in a commonly used implementation. Results of the comparison might differ when alternative settings are used. However, the aim was not to optimize the MNI normalization, but to provide a benchmark that reflects a well-known and commonly used normalization method.

In testing validity of the Cgrid approach it is assumed that the topographical organization of the sensorimotor cortex is in proportion to its shape. This means that even if the absolute location of an activity hotspot differs from one subject to another, the hotspot’s relative location—that is, the location relative to the dimensions of the sensorimotor cortex—is assumed to be the same across subjects. Likewise, this assumption applies also to the left versus the right hemisphere. Cgrid exploits this postulated relative organization of the sensorimotor cortex, and effectively places the sensorimotor cortex of each individual in a proportional space. As a result, the anatomical differences between subjects are discounted for, as well as differences between the left and right sensorimotor cortex. Subjects displayed some variations in not only the magnitude and location of activity, but also in the extent of activation along the sensorimotor cortex (compare for example the tongue activity on the right hemisphere in subjects 4 and 6). These differences may reflect variations in cortical representation, but may also well reflect differences in how tasks (even simple tasks) are performed. The calculated similarity scores are derived from Pearson correlations of the complete Cgrid pattern, and thus include areas that should not activate during the task. This makes this measure sensitive to engagement of additional body parts in a given task.

Cgrid employs several cumulative preprocessing steps that may increase the chances of biasing results for individual subjects. It is however difficult to evaluate on theoretical grounds the impact of each individual processing step and its interaction with the other steps. Similarities from Cgrid representations were compared to other methods for brain normalization, where biases should have similar effects. If individual results would be excessively biased, such bias would negatively impact the similarity across subjects, and our method would perform worse than the others, which was not the case.

Given a flattened surface reconstruction, the Cgrid method is automatic. We used Caret to generate these, which requires some manual steps, but this could be automated as well. Although the current implementation of the mapping is fully automatic, manual adjustments on the procedure may be needed in cases where the integrity of gyri and sulci is compromised, for example in patients suffering from brain atrophy or lesions. An algorithm monitoring the deviation of precentral and postcentral borders with respect to the central sulcus could be devised to notify the user if a manual adjustment is needed.

Cgrid is particularly suitable for studying activity patterns on the left and right sensorimotor cortex within subjects, and for the comparison of groups of subjects (for example healthy and diseased), as well as for longitudinal studies on for example normal development or disease-related processes, where it can be used to quantify and visualize changes in activation hotspots over time. It might be less beneficial in cases where very detailed patterns in individual subjects are studied, as transformation of these patterns could be disruptive. Advantages of Cgrid are that it provides a clear, easy to interpret and consistent representation of the sensorimotor cortex. It allows for a straightforward comparison of activation patterns between groups of subjects, but also for quantification of possible alterations (for example shifts and focality) in activation patterns in longitudinal studies, for example in the areas of development, progressive disease or plasticity (Bruurmijn et al. 2017). As the sensorimotor cortex for each individual is mapped onto the same space, Cgrid allows for comparing whole activity patterns at once, even if they consist of multiple distributed hotspots. In principle, the Cgrid approach can be extended to other primary anatomical regions, and perhaps even to associative cortex where topography is less consistent. Moreover, Cgrid allows for mapping of any cortical parameter, and can accommodate weighing of tile values by the number of included vertices to better represent their quantity where relevant.

In conclusion, we present a Cartesian representation of the anatomical sensorimotor cortex in humans, with the aim to facilitate quantitative comparisons of brain activity within and between subjects and visualize results. Results of data from 20 subjects show that the Cgrid performs equal or better than comparisons in MNI space, while carrying the benefit of enabling spatial quantitative comparisons of activity patterns.

## Information Sharing Statement

The Cgrid method has been put into a toolbox and can be downloaded from https://github.com/mathijsraemaekers/Cgrid-toolbox.

The ethics protocol limits data publication from a public repository, but does allow data sharing upon request. Please contact the corresponding author.
